# Task‐induced deactivation dysfunction during reward processing is associated with low self‐esteem in a possible subtype of major depression

**DOI:** 10.1002/brb3.3545

**Published:** 2024-06-14

**Authors:** Antonia K. Jüllig, Sandi Hebib, Helena Metzker, Eva Gruber, Oliver Gruber

**Affiliations:** ^1^ Section for Experimental Psychopathology and Neuroimaging, Department of General Psychiatry University Hospital Heidelberg Heidelberg Germany

**Keywords:** major depressive disorder, pregenual anterior cingulate, reward, self‐esteem, subtype, task‐induced deactivation

## Abstract

**Introduction:**

Low self‐esteem is a frequent symptom in major depressive disorder (MDD). This functional magnetic resonance imaging study investigated whether MDD patients with low self‐esteem show a distinct neural pathophysiology. Previous studies linked low self‐esteem to reduced task‐induced deactivation of the pregenual anterior cingulate cortex (pgACC) as a part of the default mode network, and to reduced connectivity between pgACC and reward system. Goya‐Maldonado et al. identified an MDD subtype with pgACC and ventral striatal overactivations during reward processing. We hypothesized that this subtype might be characterized by low self‐esteem.

**Methods:**

Eighty‐three MDD patients performed the desire–reason dilemma task and completed the Rosenberg Self‐Esteem Scale (RSES). Brain activity during bottom‐up reward processing was regressed upon the RSES scores, controlling for depression severity measured by the Montgomery–Åsberg Depression Rating Scale. To corroborate the findings, we compared self‐esteem scores between patient subgroups with impaired task‐induced deactivation (*n* = 31) and with preserved task‐induced deactivation (*n* = 31) of the pgACC.

**Results:**

Consistent with our a priori hypothesis, activity in a bilateral fronto‐striatal network including pgACC and ventral striatum correlated negatively with RSES scores, also when controlling for depression severity. In the additional analysis, patients with impaired task‐induced pgACC deactivation showed lower self‐esteem (*t* (52.82) = −2.27; *p* = .027, *d* = 0.58) compared to those with preserved task‐induced pgACC deactivation.

**Conclusions:**

We conclude that low self‐esteem in MDD patients is linked to a task‐induced deactivation dysfunction of the pgACC. Our findings suggest that a previously described possible subtype of MDD with pgACC and ventral striatal overactivations during reward processing is clinically characterized by low self‐esteem.

## Graphical abstract

Self‐esteem in patients with major depressive disorder (MDD) correlated negatively with reward‐related activity changes in the pregenual anterior cingulate cortex (pgACC) and the ventral striatum. This suggests that a previously described MDD subtype with reward‐related overactivations in pgACC and ventral striatum is clinically characterized by low self‐esteem.

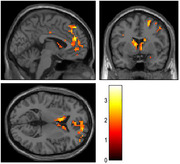

## 1 INTRODUCTION

Low self‐esteem plays a pivotal role among the symptoms of major depressive disorder (MDD). As a vulnerability factor, it predicts the development of MDD (Orth & Robins, 2013). It is also associated with a bad prognosis, enhancing the risk for suicidality (O'Neill et al., 2021; Yin et al., 2022) and undermining therapy motivation (Wood et al., 2009). Therefore, patients with pronounced feelings of inadequacy need specific support. Research is needed to investigate whether these patients form a pathophysiologically distinct subgroup that might also respond differently to treatment options than other MDD patients. The present functional magnetic resonance imaging (fMRI) study addressed this question by investigating a regressional correlation between neural activity in a reward task and self‐esteem in MDD patients.

The neural underpinnings of low self‐esteem are still poorly understood. Evidence from the few fMRI studies that have addressed low self‐esteem converges onto a central role of the pregenual anterior cingulate cortex (pgACC; Frewen et al., 2013; Wagner et al., 2013, 2015). In an emotional self‐referential processing task, Wagner et al. (2013) found enhanced pgACC activity while healthy participants read negative statements, for example, “I consider myself to be a loser,” compared to positive or neutral self‐referential statements. pgACC activity also correlated positively with the endorsement of the negative self‐descriptions as an indicator of low trait self‐esteem. Interestingly, the pgACC activation was not limited to the self‐referential processing task, but persisted during a following Stroop task. This went along with poorer cognitive control performance and reduced blood oxygenation level dependent (BOLD) responses in prefrontal brain areas following negative compared to positive or neutral self‐referential processing. In a following study on MDD patients, these findings were replicated in healthy controls while the patients showed an overactivation of the pgACC independent of the valence of the self‐referential processing. Yet, also in patients, only the negative self‐referential processing was followed by reduced prefrontal BOLD responses and performance deficits in the Stroop task (Wagner et al., 2015). Frewen et al. (2013) used a priming task to operationalize self‐esteem more implicitly. In healthy women, they found enhanced BOLD responses in the pgACC when participants associated a picture of themselves, compared to a stranger, with negative trait words. Yet, these studies have experimentally manipulated negative self‐referential processing, whereas our aim was to investigate interindividual differences in trait self‐esteem as a symptom of MDD, measured by the Rosenberg Self‐Esteem Scale (RSES; Rosenberg, 1965) as an established scale.

Chavez and Heatherton (2015, 2017) found low self‐esteem, as measured on the Janis and Field Feelings of Inadequacy Scale (Fleming & Courtney, 1984), to be associated with reduced structural and functional connectivity between a pgACC/ventromedial prefrontal cortex region and the ventral striatum in a non‐clinical sample. They interpreted this as an impaired feedback loop between self‐representations, that have been linked to medial prefrontal areas (for a meta‐analysis, see Qin & Northoff, 2011), and reward processing in the ventral striatum, so that the self‐concept cannot be associated with positive emotions (Chavez & Heatherton, 2015; Izuma et al., 2018). This raises the question whether reward processing is altered in people with low self‐esteem. In a behavioral experiment, the amount to which healthy participants used expected reward values for decision‐making correlated positively with RSES scores, indicating a reduced reward sensitivity in low self‐esteem individuals (Ogasawara et al., 2020). The neural correlates of reward system dysfunctions associated with low self‐esteem deserve further investigation.

A different branch of research has focused on the role of the pgACC in the pathophysiology of MDD and its potential subtypes. On the functions of the pgACC, a tracer study in nonhuman primates found it to be part of a circuit connecting the dorsolateral prefrontal cortex via the pgACC and subgenual anterior cingulate with subcortical structures like the amygdala and the ventral striatum, suggesting that it might play a role in emotion regulation (Joyce et al., 2020). The pgACC is also known to be a part of the default mode network (DMN; Raichle et al., 2001). In healthy people, the DMN is thought to be involved in self‐referential processing mainly during phases of rest and is inhibited during tasks that require attention to external stimuli (Gusnard et al., 2001). Previous fMRI studies found this task‐induced deactivation to be impaired in MDD patients in a variety of tasks, such as the n‐back working memory task (Bartova et al., 2015; Rodriguez‐Cano et al., 2017), executive control paradigms like the Stroop task (Wagner et al., 2006), or the multi‐source interference task (Davey et al., 2012), and emotional evaluation tasks (Grimm et al., 2009, 2011; Walter et al., 2009; Zhang et al., 2017). For reward tasks, evidence on task‐induced deactivation deficits in MDD is still missing. Zhang et al. (2017) give a hint on this, as they found enhanced medial prefrontal cortex activity in MDD patients compared to controls selectively during cueing of positively valenced pictures.

There is ample evidence that not only pgACC overactivation is a marker of MDD, but that individual differences in pgACC functioning between MDD patients might also predict treatment response, giving a hint to possible neurobiological subtypes (Jamieson et al., 2021; Weigand et al., 2022; Whitton et al., 2019; for a review, see Pizzagalli, 2011). High pgACC metabolism (Mayberg et al., 1997) and electroencephalographic activity (Korb et al., 2011; Pizzagalli et al., 2001, 2018) in the resting state have been associated with good responses to different antidepressant treatments. However, the role of high pgACC activity during tasks in the sense of missing task‐induced deactivation is less clear. Davey et al. (2021) found task‐induced pgACC deactivation to be prognostic for remission, while Godlewska et al. (2018) observed better responses to SSRI in patients with high pgACC activity during emotional picture viewing. Further characterizing the different groups of MDD patients with high and low task‐induced pgACC deactivation, also regarding clinical symptoms, might help to resolve this issue.

Goya‐Maldonado et al. (2015) identified a subgroup of MDD patients with enhanced activity in the pgACC and ventral striatum during bottom‐up processing and top‐down suppression of immediate rewards in the desire–reason dilemma (DRD) task (Diekhof & Gruber, 2010). This suggests an impaired feedback loop between pgACC and ventral striatum as a pathomechanism in a part of MDD patients, resembling the pattern that Chavez and Heatherton (2015) found for individuals with low self‐esteem. Goya‐Maldonado et al. did not find any clinical differences between the high‐activating and a second, low‐activating subgroup, yet did not investigate self‐esteem. Combining the evidence for pgACC overactivation as a neural correlate of low self‐esteem on the one hand and as a feature of an MDD subgroup on the other hand led us to the question whether the “high‐activating” (Goya‐Maldonado et al., 2015) subgroup is characterized by low self‐esteem.

To clarify this, we conducted an fMRI study using the DRD task in a sample of MDD patients in whom we assessed self‐esteem with the RSES. We hypothesized that BOLD responses in the pgACC and ventral striatum would be higher in patients with lower self‐esteem, that is, would correlate negatively with RSES scores. To corroborate findings, we also investigated whether patients with impaired or preserved task‐induced deactivations in the pgACC differ in self‐esteem.

## 2 MATERIALS AND METHODS

### 2.1 Participants

The cases included in this study were selected from a database from a larger project, containing fMRI data in several paradigms from patients of the University Hospital Heidelberg. Patients included in this database were diagnosed with MDD, bipolar disorder, or schizophrenia confirmed by a clinical interview the day before scanning, were free from acute suicidality, substance dependence, neurological or metabolic diseases, had normal or corrected‐to‐normal vision and normal color vision, and gave written informed consent for data use in research. Participants were paid 20€ plus a bonus up to 20€ according to task performance.

For this study, we selected patients diagnosed with MDD for whom complete questionnaire data in the RSES and the Montgomery–Åsberg Depression Rating Scale (MADRS) as well as fMRI data in the DRD paradigm were available. This sample consisted of 83 patients (46 female, 37 male) aged 18–61 years (*M* = 34.46; *SD* = 10.84).

### 2.2 Reward task

**FIGURE 1:**
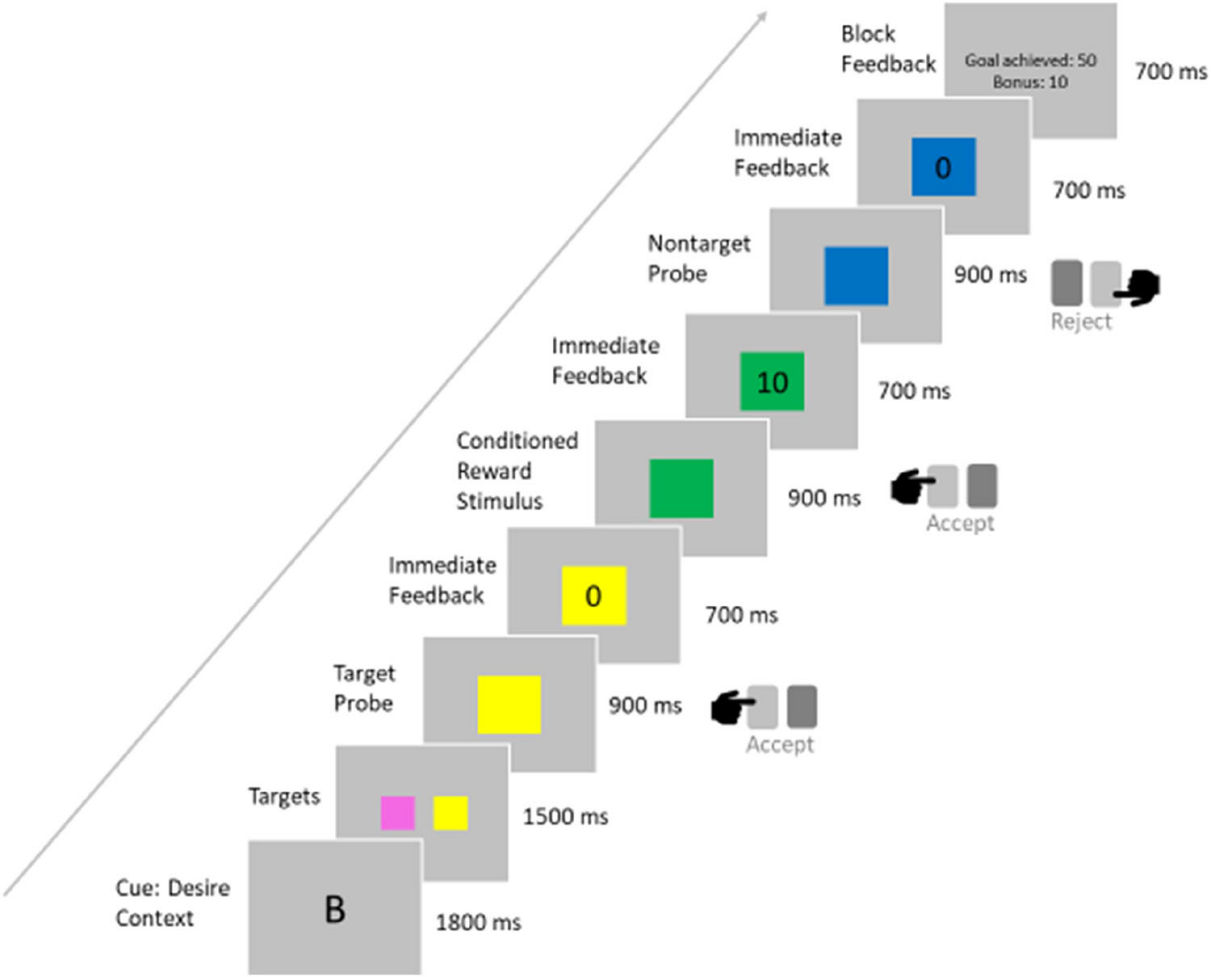
Example stimulus presentation sequence for a block of the desire context of the desire–reason dilemma paradigm.

We used the DRD paradigm (Diekhof & Gruber, 2010), a sequential forced‐choice task which allows to investigate bottom‐up activation of the reward system in immediately rewarded decisions as well as its top‐down modulation when immediate reward is forgone to achieve long‐term goals. Unlike other widely used reward tasks, for example, the monetary incentive delay task, the DRD task does not include a delay phase and thus avoids confounds with anticipatory processes that might be impaired in depressed patients. To prepare for this task, participants underwent a session of operant conditioning the day before scanning, where they learned by trial and error that two out of six colors presented in random order were associated with immediate reward when accepted by keypress (for details, see Diekhof & Gruber, 2010). In the actual task, these two “bonus colors” were presented again in two different contexts: the desire context (DC) and the reason context (RC). In both contexts, a superordinate goal had to be pursued: two colors (blue, yellow, turquoise, or pink) were cued as target colors in the beginning of a block. Then, while colored squares were presented in rapid succession, the task was to accept the target colors by pressing the left key and to reject all others by pressing the right key. This led to the feedback “goal achieved” at the end of a block and a bonus of 50 points added to a score that was converted into a monetary reward after the experiment. In the DC, additionally, the two conditioned bonus colors (red and green) could be accepted, leading to an immediate feedback about a reward of 10 points added to the score, so, reacting to immediate rewards was beneficial for the overall outcome. In the RC, however, accepting the conditioned colors terminated the block with the feedback “goal failed,” so that immediate rewards had to be suppressed in favor of the long‐term goal and a dilemma between the conditioned desire and the “reasonable” goal pursuit occurred when a conditioned color was presented. For our analyses, we focused on the desire context. An example trial sequence with stimulus timings is shown in Figure 1.

### 2.3 Self‐esteem and depression scales

Self‐esteem was assessed using the RSES (Rosenberg, 1965) in the German version by Von Collani and Herzberg (2003), a 10‐item self‐rating questionnaire. Depression severity was measured by the German version of the MADRS (Montgomery & Åsberg, 1979; Schmidtke et al., 1988).

### 2.4 fMRI acquisition

For the first 20 patients, we acquired fMRI data on a 3 Tesla MAGNETOM TrioTim syngo MR B17 (Siemens, Erlangen, Germany) at the Neuroradiology Department of the University Hospital Heidelberg, Germany. This scanner was upgraded to a 3 Tesla MAGNETOM TIM TRIO PRISMA FIT (Siemens, Erlangen, Germany) on which we acquired data for the following 63 patients. For all participants, we used a 20‐channel head coil and an EPI sequence (TR, 1900 ms; TE, 30 ms; flip angle, 70°; FOV, 192 mm^2^; 64 slices in interleaved order; EPI‐factor, 64; 3 mm isotropic voxels). Structural T1‐weighted images were additionally acquired for each participant (TR, 2250 ms; TE, 3.26 ms; flip angle, 9°; FOV, 256 mm^2^; 1 mm isotropic voxels).

### 2.5 fMRI pre‐processing and analysis

All fMRI data processing was performed in SPM12 (https://www.fil.ion.ucl.ac.uk/spm/software/spm12/). For pre‐processing, the data were motion corrected by realignment and unwarping, slice time corrected, normalized to the Montreal Neurological Institute (MNI) 3 mm standard template, and smoothed with a 6 mm FMWH Gaussian kernel.

Based on previous findings of high‐ and low‐ activating MDD subgroups (Goya‐Maldonado et al., 2015), we hypothesized that low self‐esteem was positively correlated with bottom‐up activation of the reward system. Therefore, on single‐subject level, we contrasted the presentation of the conditioned bonus color in the DC against implicit baseline. Our whole general linear model contained 11 regressors for the different experimental phases convolved with a canonical hemodynamic response function (HRF), that is, the two context conditions DC and RC, target, bonus, and neutral colors for each context, cue, feedback, and termination, with all regressors except the bonus color in DC set to 0.

On group level, we correlated this BOLD response during presentation of the conditioned reward stimulus with the RSES total scores as an indicator of self‐esteem in a regression model. As we were interested in brain activity associated with low self‐esteem, we defined a t‐contrast for a negative correlation between RSES score and BOLD response. To control for the severity of depressive symptoms other than self‐esteem, we included the MADRS scores as covariate of no interest. To this end, we removed the closely self‐esteem‐related item 9 (pessimistic thoughts, including feelings of inferiority and self‐reproach) from the total MADRS score. The fMRI analyses were performed on the whole brain, using a voxel‐wise significance threshold of *p *= .05 (uncorrected) with a minimum cluster size of about 10 voxels as an initial search criterion. Subsequently, standard corrections for multiple comparisons were applied including small‐volume corrections for brain regions with clear a priori hypotheses.

To gain further evidence on whether MDD patients with and without dysfunctions in task‐induced deactivation differ in self‐esteem, we conducted an additional subgroup analysis focusing on the pgACC. Assuming that task‐induced deactivation is a healthy neurophysiological mechanism whose dysfunction is linked to the symptom of low self‐esteem, we aimed at quantifying this dysfunction in our patient sample. To this end, we first identified in a separate, representative healthy control sample from our database, consisting of the best performers in the DRD task (*n *= 30; 15 female, 15 male, age: *M* = 24.60 years; *SD* = 2.03 years), the pgACC voxel with the strongest effect of task‐induced deactivation during presentation of conditioned reward stimuli in the DC. Then, for the whole patient sample, we extracted the *T* values from this voxel, located at the MNI coordinates −3 57 −6, and used them to identify two extreme groups. As a subgroup with impaired task‐induced pgACC deactivation (“non‐deactivators”), we defined patients without significant (*α* = 0.05) deactivation, that is, with *T* values not smaller than *t* = −1.67. For comparison, we defined an equally large (*n* = 31) extreme group with maximum deactivations in this target pgACC voxel. We compared mean RSES scores between the two groups, controlling for group differences in age, sex, and depression severity. Supplementarily, we transformed the RSES scores into self‐esteem levels (García et al., 2018; Rosenberg, 1965) and compared their frequencies between the subgroups.

## 3 RESULTS

### 3.1 Self‐esteem and depression scores

Overall, participants had a mean RSES score of 13.45 (*SD* = 5.80, *Range =* 3–30) points with the scale ranging from 0 to 30 points, corresponding to a low self‐esteem level, and a mean MADRS score of 21.23 (*SD* = 8.07, *Range *= 3–45) points, corresponding to a moderate depressive syndrome (Montgomery & Åsberg, 1979; Neumann & Schulte, 1989).

### 3.2 BOLD responses during reward processing

As a manipulation check, we ran a one‐sample *t*‐test comparing reward collection in the DC against baseline. In line with previous studies using the DRD paradigm (Diekhof & Gruber, 2010; Richter et al., 2015; Trost et al., 2014), it showed strong activations in the bilateral ventral striatum (left: −15 6 6; *t* = 6.54, right: 12 6 3; *t* = 7.41) and other regions of the extended reward system, that is, the anterior ventral prefrontal cortex, frontomedian cortex, and anterior insula. Unexpectedly, we did not find any activation in the ventral tegmental area. This may be due to hypoactivations in a part of MDD patients (Goya‐Maldonado et al., 2015) or more generally due to problems with signal‐to‐noise ratios in this region.

### 3.3 BOLD responses with negative correlation to self‐esteem scores

**FIGURE 2:**
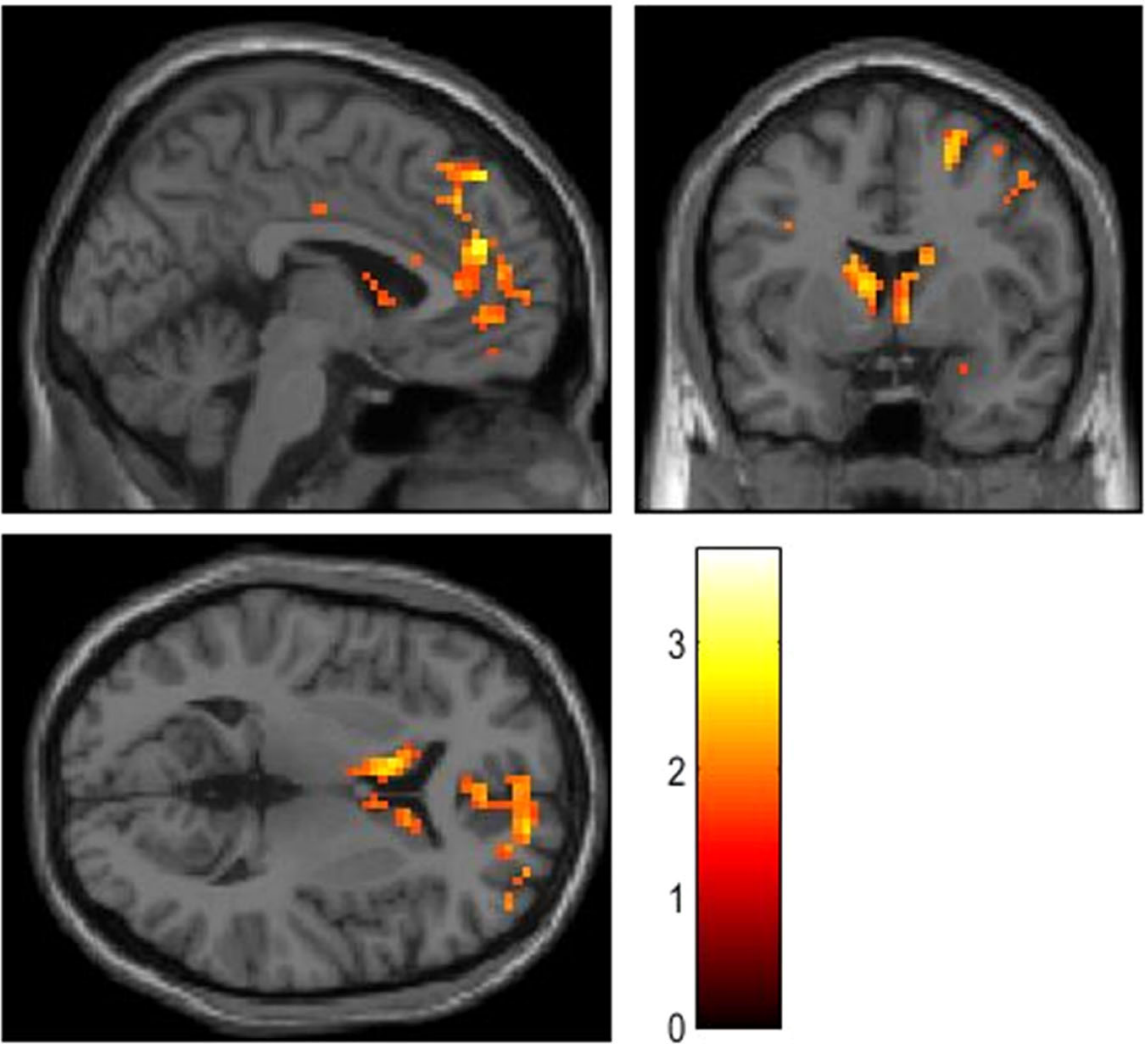
Reward‐related brain activity negatively correlated with self‐esteem scores in major depressive disorder (MDD) patients. Increased reduced blood oxygenation level dependent responses in this fronto(median)‐striatal network during presentation of conditioned reward stimuli correlated with reduced self‐esteem scores in MDD patients. To increase visibility of the involved brain regions, the statistical threshold of the t‐map was lowered to *p* < .05, uncorrected. See Table 1 for statistical details.

We were interested in brain activity associated with reduced self‐esteem, particularly expecting higher activity in the pgACC and ventral striatum (Goya‐Maldonado et al., 2015) to be associated with lower self‐esteem. In our model, we regressed RSES scores upon the BOLD responses during reward presentation in the DC of the DRD paradigm. Confirming our a priori hypothesis, RSES scores were negatively correlated with activity in an extended bilateral fronto‐striatal network including pregenual anterior cingulate and adjacent frontomedian cortices as well as ventral and dorsal striatum (Table 1 and Figure 2). The extended pregenual/frontomedian cluster reached a statistical trend level even when using overly conservative correction for the whole brain according to both false discovery rates (*p* = .09) and family‐wise errors (*p* = .095). Small‐volume corrections using spheres with a radius of 5 mm around a priori coordinates taken from the literature (Trost et al., 2014) were performed for brain regions with clear a priori hypotheses (pregenual ACC and ventral striatum) but failed to reach the level of statistical significance. However, important to note, this was not due to small effect sizes, as these would have been sufficiently high for surviving this established correction for multiple comparisons both in the pregenual ACC and in the striatum. Instead, the reason for this failure to reach statistical significance at this level of correction was a moderate anatomical displacement of the effects in the present study as compared to the exact a priori coordinates with maximum activation reported in the reference study. In other words, the sufficiently strong effects in our study lay slightly outside the small‐volume sphere used for correction. This may have occurred due to methodological limitations, for example, of anatomical precision of normalization procedures used in standard processing of fMRI data.

**TABLE 1:** Brain activity negatively correlated with self‐esteem scores in major depressive disorder patients during reward processing. Shown are the brain regions whose peak‐level reduced blood oxygenation level dependent responses to presentation of conditioned reward stimuli correlated negatively with Rosenberg Self‐Esteem Scale scores. To avoid false negative results, we included all brain regions reaching a minimum *p* < .01 with a minimum cluster size of about 10 voxels. Asterisks represent the following significance thresholds: *statistical trend at *p* (false‐discovery rate (FDR) and family‐wise error (FWE) corrected for the whole brain) < .1; **significant at voxel‐wise *p* (uncorrected) < .005. The effects in brain regions indicated in bold were independent of general depression severity as confirmed in a control analysis (see Section 3.4 and Table S1).

Brain region	MNI coordinates and *T* values	Cluster size (number of voxels)
**R dorsal frontomedian cortex**	3 42 45 (3.72)**	602*
**L dorsal pregenual anterior cingulate cortex**	−3 42 21 (2.95)**	602*
R inferior frontal junction area	48 18 39 (2.92)	602*
L dorsal frontomedian cortex	−6 42 45 (2.89)**	602*
**R anteroventral prefrontal cortex/pregenual anterior cingulate cortex**	15 60 3 (2.83)**	602*
**R dorsal pregenual anterior cingulate cortex**	9 36 21 (2.78)**	602*
L pregenual anterior cingulate cortex	−6 60 15 (2.62)	602*
R middle frontal gyrus	36 21 54 (2.46)	602*
**L subgenual anterior cingulate cortex**	−9 39 −12 (2.01)	602*
L anterior middle frontal gyrus/superior frontal sulcus	−18 39 36 (2.74)**	35
L anterior middle frontal gyrus/superior frontal sulcus	−18 27 51 (2.29)	14
**L ventral striatum**	−6 12 3 (2.71)**	76
**L ventral/dorsal striatum**	−9 6 9 (2.57)	76
**L dorsal striatum**	−12 6 15 (2.36)	76
**R ventral striatum**	6 9 0 (2.61)**	52
R superior frontal sulcus	21 6 57 (2.47)	27
Posterior cingulate cortex	0 −12 36 (2.46)	37
L superior frontal gyrus	−18 27 51 (2.29)	14
**R dorsal striatum**	12 6 18 (2.22)	8
L inferior frontal junction/dorsal Broca's area	−45 15 21 (2.15)	9
**L middle temporal gyrus, middle third**	−48 −33 −6 (2.14)	9

### 3.4 Control for depression severity

RSES scores were significantly negatively correlated with the severity of other depressive symptoms measured by MADRS scores (*r* = −0.56; *p* < .001). To control for this possible confound, we repeated our fMRI analysis adding the MADRS scores to the multiple regression model as a covariate of no interest. This control analysis confirmed that particularly the effects revealed in our a priori regions of interest (pregenual ACC and striatum) did not result from a more general association with other symptoms of depression but were indeed specifically related to reduced self‐esteem scores (cf. Table 1 and Table S1, brain regions indicated in bold).

### 3.5 BOLD responses with positive correlation to self‐esteem scores

In order to provide a more comprehensive overview of the relationship between changes in reward‐related brain activity and self‐esteem in MDD patients, we also conducted an exploratory analysis of BOLD responses with positive correlation to self‐esteem scores although there was no a priori hypothesis for brain regions showing this type of response. This analysis revealed positive correlations with RSES scores predominantly in posterior brain regions including occipito‐temporal areas of the so‐called ventral visual pathway, (intra)parietal and intraoccipital cortices as well as posterior insular and cerebellar regions (Figure S2A). Thus, when directly comparing this pattern of positive correlations with the pattern of negative correlations with RSES scores (Figure S2B), this analysis provides first preliminary evidence for a dysbalance of brain activity in MDD patients with reduced self‐esteem in terms of anterior frontal hyperactivations versus posterior parietal and occipito‐temporal hypoactivations.

### 3.6 Corroboration of the main finding by means of subgroup analysis

Although the main finding from the regression analyses reported in Sections 3.3 and 3.4, that is, an association between symptoms of reduced self‐esteem in MDD patients and pregenual ACC (hyper)activity in these patients is well in line with the a priori hypothesis of this study, one may criticize the moderate significance levels of these results. For this reason, in order to provide further evidence for this relationship, we performed additional analyses using a different and complementary approach, namely a specific subgroup analysis. This analysis aimed at investigating whether MDD patients with impaired task‐induced deactivation of the pgACC differ from MDD patients with preserved task‐induced deactivation of the pregenual ACC in terms of self‐esteem scores (see Methods for details). Since variances differed between the groups, we ran a nonparametric two‐sided Welch test to compare mean RSES scores between patients without significant deactivation and those with maximum deactivation of the pgACC. The non‐deactivator subgroup showed significantly lower self‐esteem (*M* = 11.10, *SD* = 4.32) compared to the group with maximum deactivations (*M* = 14.23, *SD* = 6.36; *t* (52.82) = −2.27; *p* = .027, *d* = 0.58) with the significance level set to *α* = 0.05. A *χ* ^2^‐test on the frequencies of the self‐esteem levels confirmed that the non‐deactivators showed a low level of self‐esteem significantly more often than expected by chance (*χ* ^2^ = 9.37; *p* = .009).

We controlled for depression severity differences comparing the MADRS scores between the subgroups after removing a self‐esteem relevant item (item 9, see methods section 2.5). These depressive symptoms other than self‐esteem were descriptively higher in non‐deactivators with a mean MADRS score of 20.68 (*SD* = 5.11) compared to the maximum deactivation subgroup (*M* = 17.65; *SD* = 9.69). Yet, this difference was not statistically significant, and Bayesian *t*‐tests remained inconclusive (*t* (45.48) = 1.54; *p* = .130; *BF*01 = 1.43). The subgroups did not differ significantly in age (non‐deactivators: *M* = 37.10, *SD* = 11.43; maximum deactivators: *M* = 33.90, *SD* = 11.47; *t* (60) = −1.10; *p* = .277, *BF*01 = 2.33) or sex distribution (non‐deactivators: 19 female, 12 male; maximum deactivators: 14 female, 17 male; *χ* ^2^ = 1.62; *p* = .203; *BF*01 = 1.48).

## 4 DISCUSSION

The present study aimed at investigating whether MDD patients with low self‐esteem show distinct neural correlates and thus might form a pathophysiological MDD subtype. Previous studies linked low self‐esteem to high activity of the pgACC during tasks (Frewen et al., 2013; Wagner et al., 2013, 2015) when it is usually deactivated as a part of the DMN (Gusnard et al., 2001; Raichle et al., 2001), and to an impaired communication between the mPFC/pgACC and the ventral striatum (Chavez & Heatherton, 2015). Based on this, we were interested in whether an MDD subtype with overactivations in the pgACC and ventral striatum during bottom‐up reward processing previously described by Goya‐Maldonado et al. (2015) was clinically characterized by low self‐esteem. In our regressional correlation between self‐esteem scores and BOLD responses during reward presentation in the DRD task, we therefore expected low self‐esteem to go along with high activity in the pgACC and ventral striatum. Confirming our hypothesis, we found a negative correlation between RSES scores and BOLD responses in the bilateral dorsal pgACC and the right ventral striatum. This also held when we added the MADRS scores as a covariate of no interest and was thus independent of depression severity.

Interestingly, the region we found in the dorsal pgACC (MNI coordinates: left −6 42 21, right 6 36 21) lies closely adjacent to an area that Sheline et al. (2010) termed the dorsal nexus (MNI coordinates: left −24 35 28, right 18 34 29). In their resting‐state fMRI functional connectivity analysis, Sheline et al. described the dorsal nexus as a hub connecting three networks: the DMN, the fronto‐parietal cognitive control network, or “task‐positive network” (TPN; Hamilton et al., 2011) and the affective network comprising pre‐ and subgenual anterior cingulate areas probably connected to emotion‐processing subcortical structures like the amygdala and the ventral striatum (Joyce et al., 2020). Sheline et al. found enhanced connectivity between the dorsal nexus and all these three networks, especially the DMN, in MDD patients compared to healthy controls. In the light of this framework, one might speculate that, in the present study, if MDD patients with low self‐esteem lack DMN deactivation during the task (Wagner et al., 2013, 2015), but the DRD task requires activity of the TPN (Richter & Gruber, 2017; Richter et al., 2015; Trost et al., 2014, 2016) which is modeled as an opponent of the DMN (Hamilton et al., 2011), it is more effortful for the dorsal nexus to coordinate the activity of the three networks, reflected in an enhanced BOLD response. This should be further investigated using, for example, psychophysical interaction (PPI) analysis.

To unambiguously interpret our finding in the pgACC, it is important to consider that, in a coordinate based meta‐analysis, Acikalin et al. (2017) found the DMN to overlap in the vmPFC with a network processing subjective value. The pgACC has also been described to be involved in the representation of reward values in decision‐making tasks (Rupprechter et al., 2021; for a meta‐analysis, see Bartra et al., 2013). Does pgACC activity in our reward task therefore actually reflect deficits in task‐induced DMN deactivation, or might it be alternatively explained by reward‐related activation? Our additional subgroup analysis helps to disentangle these possibilities as, here, we specifically investigated task‐induced deactivation: we chose a pgACC voxel of interest (VOI) that was maximally deactivated during reward presentation in the DRD paradigm in a healthy control sample. Then, we compared self‐esteem scores between two extreme groups from our MDD patient sample: a group that lacked significant deactivation in the VOI, and an equally large group with the highest deactivations. We confirmed our hypothesis that patients without pgACC deactivation showed significantly lower self‐esteem scores than those with high pgACC deactivation. Furthermore, previous studies on the DRD paradigm speak against the alternative explanation since the pgACC was not among the regions typically activated during reward presentation (Diekhof & Gruber, 2010; Richter et al., 2015; Trost et al., 2014). Thus, our results provide further evidence for a link between dysfunctional task‐induced pgACC deactivation and feelings of inadequacy in MDD patients (Wagner et al., 2015).

Although it is important to distinguish DMN activity and reward processing in our paradigm, we were interested in the interplay between these two systems (Dobryakova & Smith, 2022) that is known to be altered in MDD (Ding et al., 2022; Rupprechter et al., 2020). Along with pgACC activity, we found activity in the ventral striatum to correlate negatively with self‐esteem. This matches the pattern that Goya‐Maldonado et al. (2015) observed in their “high‐activating” MDD subtype. As it persisted during the reason context of the DRD task when ventral striatal reactions to conditioned rewards should be suppressed, they interpreted it as a top‐down modulation dysfunction of the reward system (Goya‐Maldonado et al., 2015). On this top‐down modulation, Ferenczi et al. (2016) conducted an animal study combining optogenetics with fMRI in awake rats. They observed that excitatory optogenetic stimulation of dopaminergic neurons in the ventral striatum during fMRI enhanced BOLD responses in the ventral striatum. This reaction was reduced when, at the same time, mPFC excitability was optogenetically enhanced. mPFC stimulation also reduced the rats’ reward‐seeking behavior. The authors conclude that the mPFC exerts inhibitory top‐down control onto the ventral striatum, that is, mPFC activation inhibits ventral striatal responses to reward. At first glance, this seems to contradict our results: according to Ferenczi et al., if the activity of the pgACC, a part of the mPFC, is enhanced in people with low self‐esteem, they should show reduced ventral striatal activity at reward presentation. Instead, BOLD responses in both the pgACC and the ventral striatum were stronger the lower self‐esteem was. Several reasons might explain these differences. First, it is unclear whether the mPFC region that Ferenczi et al. stimulated corresponds to the pgACC in humans. Second, optogenetic stimulation is limited to a short time window, whereas we would expect rather persisting DMN activation in humans with low trait self‐esteem. Ferenczi et al. accounted for this by using an optogenetic method that did not lead to immediate firing of the mPFC neurons, but to a long‐term increase in excitability. Yet, their fMRI experiment required to switch stimulation on and off within seconds and therefore might not capture long‐term responses to enhanced mPFC excitability, for example, changes in connectivity, or compensatory regulations. Furthermore, Ferenczi et al. stimulated ventral striatal dopaminergic neurons that code for reward expectation. Our DRD paradigm, however, is designed to investigate the processing of immediate rewards and deliberately excludes anticipatory processes like expectation, so it might activate different ventral striatal neurons (for a review, see Schultz et al., 2006). Most importantly, Ferenczi et al. investigated physiological processes in healthy animals, not a model of MDD. In healthy humans, reward has been found to strengthen functional connectivity between DMN and ventral striatum (Dobryakova & Smith, 2022). In MDD patients (Ding et al., 2022; Rupprechter et al., 2020) and people with low self‐esteem (Chavez & Heatherton, 2015, 2017), however, there are hints on weakened functional and structural connections possibly preventing the pgACC signal from reaching the ventral striatum. Therefore, the inhibitory top‐down influence from the pgACC to the ventral striatum might be the physiological mechanism, while our results suggest that this is impaired in an MDD subtype (Goya‐Maldonado et al., 2015) characterized by low self‐esteem. Understanding how self‐esteem‐related dysfunctions in task‐induced pgACC deactivation interact with reward processing might help to clarify in future studies why low self‐esteem acts as an MDD vulnerability factor (Orth & Robins, 2013) and how it might entail other symptoms like anhedonia (Walter et al., 2009).

As a possible limitation of our study, behavioral performance in the DRD task might covary with self‐esteem and might also be responsible for BOLD response differences. A link between stronger task‐induced DMN deactivation and better behavioral performance has been described, for example, for memory tasks (for a review, see Acikalin et al., 2017). Yet, neither the overall performance scores (*r *< 0.01, *p* = .981, *BF*01 = 11.49) nor the number of bonus points for accepting conditioned rewards (*r *= 0.04, *p* = .742, *BF*01 = 9.43) that our patients achieved in the DRD task showed significant correlations with the RSES scores, and Bayes factors supported the null hypothesis, so that this alternative explanation seems very unlikely.

## 5 CONCLUSIONS

Taken together, we found that impaired task‐induced deactivation of the pgACC in a reward task was associated with low self‐esteem in MDD patients, also when controlling for depression severity. The pattern of BOLD responses in the pgACC and the ventral striatum, which negatively correlated with self‐esteem, matches a previously described “high‐activating” subtype of MDD (Goya‐Maldonado et al., 2015), suggesting that this possible subtype is clinically characterized by low self‐esteem. Characterizing an MDD subtype neurobiologically and clinically enables clinicians to draw inferences from symptoms about underlying neural mechanisms. Further research is needed on whether these neural correlates may qualify as biomarkers to predict responses to specific treatment options and to stratify treatment of MDD.

## AUTHOR CONTRIBUTIONS

**Antonia K. Jüllig**: Conceptualization; data curation; formal analysis; investigation; methodology; writing—original draft. **Sandi Hebib**: Data curation; investigation; writing—review and editing. **Helena Metzker**: Data curation; investigation; writing—review and editing. **Eva Gruber**: Data curation; investigation; project administration; writing—review and editing. **Oliver Gruber**: Conceptualization; data curation; formal analysis; methodology; project administration; supervision; writing—review and editing.

## FUNDING

No funding was received for conducting this study.

## CONFLICT OF INTEREST STATEMENT

There are no commercial or financial involvements which might represent a conflict of interest.

### PARTICIPANTS

Participants were informed about the purpose, the nature, and possible consequences or potential risks of the study and gave written informed consent.

### PEER REVIEW

The peer review history for this article is available at https://publons.com/publon/10.1002/brb3.3545

## Supporting information

Supporting information

Supporting information

## Data Availability

The data that support the findings of this study are available on reasonable request from the corresponding author. The data are not publicly available due to privacy or ethical restrictions.

## References

[brb33545-bib-0001] Acikalin, M. Y. , Gorgolewski, K. J. , & Poldrack, R. A. (2017). A coordinate‐based metaanalysis of overlaps in regional specialization and functional connectivity across subjective value and default mode networks. Frontiers in Neuroscience, 11, 1. 10.3389/fnins.2017.00001 28154520 PMC5243799

[brb33545-bib-0003] Bartova, L. , Meyer, B. M. , Diers, K. , Rabl, U. , Scharinger, C. , Popovic, A. , Pail, G. , Kalcher, K. , Boubela, R. N. , Huemer, J. , Mandorfer, D. , Windischberger, C. , Sitte, H. H. , Kasper, S. , Praschak‐Rieder, N. , Moser, E. , Brocke, B. , & Pezawas, L. (2015). Reduced default mode network suppression during a working memory task in remitted major depression. Journal of Psychiatric Research, 64, 9–18. 10.1016/j.jpsychires.2015.02.025 25801734 PMC4415908

[brb33545-bib-0004] Bartra, O. , McGuire, J. T. , & Kable, J. W. (2013). The valuation system: A coordinate‐based meta‐analysis of BOLD fMRI experiments examining neural correlates of subjective value. Neuroimage, 76, 412–427. 10.1016/j.neuroimage.2013.02.063 23507394 PMC3756836

[brb33545-bib-0006] Chavez, R. S. , & Heatherton, T. F. (2015). Multimodal frontostriatal connectivity underlies individual differences in self‐esteem. Social Cognitive and Affective Neuroscience, 10, 364–370. 10.1093/scan/nsu063 24795440 PMC4350482

[brb33545-bib-0007] Chavez, R. S. , & Heatherton, T. F. (2017). Structural integrity of frontostriatal connections predicts longitudinal changes in self‐esteem. Social Neuroscience, 12, 280–286. 10.1080/17470919.2016.1164753 26966986 PMC5047852

[brb33545-bib-0008] Davey, C. G. , Cearns, M. , Jamieson, A. , & Harrison, B. J. (2021). Suppressed activity of the rostral anterior cingulate cortex as a biomarker for depression remission. Psychological Medicine, 53(6), 1–8. 10.1017/S0033291721004323 36762975 PMC10123826

[brb33545-bib-0009] Davey, C. G. , Yücel, M. , Allen, N. B. , & Harrison, B. J. (2012). Task‐related deactivation and functional connectivity of the subgenual cingulate cortex in major depressive disorder. Frontiers in Psychiatry, 3, 14. 10.3389/fpsyt.2012.00014 22403553 PMC3289045

[brb33545-bib-0010] Diekhof, E. K. , & Gruber, O. (2010). When desire collides with reason: Functional interactions between anteroventral prefrontal cortex and nucleus accumbens underlie the human ability to resist impulsive desires. The Journal of Neuroscience, 30, 1488–1493. 10.1523/JNEUROSCI.4690-09.2010 20107076 PMC6633806

[brb33545-bib-0011] Ding, Y.‐D. , Chen, X. , Chen, Z.‐B. , Li, L. , Li, X.‐Y. , Castellanos, F. C. , Bai, T.‐J. , Bo, Q.‐J. , Cao, J. , Chang, Z.‐K. , Chen, G.‐M. , Chen, N.‐X. , Chen, W. , Cheng, C. , Cheng, Y.‐Q. , Cui, X.‐L. , Duan, J. , Fang, Y.‐R. , Gong, Q.‐Y. , … Guo, W.‐B. (2022). Reduced nucleus accumbens functional connectivity in rewardnetwork and default mode network in patients with recurrent major depressive disorder. Translational Psychiatry, 12, 236. 10.1038/s41398-022-01995-x 35668086 PMC9170720

[brb33545-bib-0012] Dobryakowa, E. , & Smith, D. V. (2022). Reward enhances connectivity between the ventral striatum and the default mode network. Neuroimage, 258, 119398. 10.1016/j.neuroimage.2022.119398 35724856 PMC9343171

[brb33545-bib-0013] Ferenczi, E. A. , Zalocusky, K. A. , Liston, C. , Grosenick, L. , Warden, M. R. , Amatya, D. , Katovich, K. , Mehta, H. , Patenaude, B. , Ramakrishnan, C. , Kalanithi, P. , Etkin, A. , Knutson, B. , Glover, G. H. , & Deisseroth, K. (2016). Prefrontal cortical regulation of brainwide circuit dynamics and reward‐related behavior. Science, 351, aac9698. 10.1126/science.aac9698 26722001 PMC4772156

[brb33545-bib-0014] Fleming, J. S. , & Courtney, B. E. (1984). The dimensionality of self‐esteem: II. Hierarchical facet model for revised measurement scales. Journal of Personality and Social Psychology, 46, 404–421. 10.1037/0022-3514.46.2.404

[brb33545-bib-0015] Frewen, P. A. , Lundberg, E. , Brimson‐Théberge, M. , & Théberge, J. (2013). Neuroimaging self‐esteem: A fMRI study of individual differences in women. Social Cognitive and Affective Neuroscience, 8, 546–555. 10.1093/scan/nss032 22403154 PMC3682439

[brb33545-bib-0016] Godlewska, B. R. , Browning, M. , Norbury, R. , Igoumenou, A. , Cowen, P. J. , & Harmer, C. J. (2018). Predicting treatment response in depression: The role of anterior cingulate cortex. International Journal of Neuropsychopharmacology, 21, 988–996. 10.1093/ijnp/pyy069 30124867 PMC6209854

[brb33545-bib-0017] Goya‐Maldonado, R. , Weber, K. , Trost, S. , Diekhof, E. , Keil, M. , Dechent, P. , & Gruber, O. (2015). Dissociating pathomechanisms of depression with fMRI: Bottom‐up or top‐down dysfunctions of the reward system. European Archives of Psychiatry and Clinical Neuroscience, 265, 57–66. 10.1007/s00406-014-0552-2 25327829

[brb33545-bib-0018] Grimm, S. , Boesiger, P. , Beck, J. , Schuepbach, D. , Bermpohl, F. , Walter, M. , Ernst, J. , Hell, D. , Boeker, H. , & Northoff, G. (2009). Altered negative BOLD responses in the default‐mode network during emotion processing in depressed subjects. Neuropsychopharmacology, 34, 932–943. 10.1038/npp.2008.81 18536699

[brb33545-bib-0019] Grimm, S. , Ernst, J. , Boesiger, P. , Schuepbach, D. , Boeker, H. , & Northoff, G. (2011). Reduced negative BOLD responses in the default‐mode network and increased self‐focus in depression. The World Journal of Biological Psychiatry, 12, 627–637. 10.3109/15622975.2010.545145 21247256

[brb33545-bib-0020] Gusnard, D. A. , Akbudak, E. , Shulman, G. L. , & Raichle, M. E. (2001). Medial prefrontal cortex and self‐referential mental activity: Relation to a default mode of brain function. Proceedings of the National Academy of Sciences of the United States of America, 98, 4259–4264. 10.1073/pnas.071043098 11259662 PMC31213

[brb33545-bib-0021] Hamilton, J. P. , Furman, D. J. , Chang, C. , Thomason, M. E. , Dennis, E. , & Gotlib, I. H. (2011). Default‐mode and task‐positive network activity in major depressive disorder: Implications for adaptive and maladaptive rumination. Biological Psychiatry, 70, 327–333. 10.1016/j.biopsych.2011.02.003 21459364 PMC3144981

[brb33545-bib-0022] Izuma, K. , Kennedy, K. , Fitzjohn, A. , Sedikides, C. , & Shibata, K. (2018). Neural activity in the reward‐related brain regions predicts implicit self‐esteem: A novel validity test of psychological measures using neuroimaging. Journal of Personality and Social Psychology, 114, 343–357. 10.1037/pspa0000114 29461079

[brb33545-bib-0023] Jamieson, A. , Harrison, B. J. , Razi, A. , & Davey, C. G. (2021). Rostral anterior cingulate network effective connectivity in depressed adolescents and associations with treatment response in a randomized controlled trial. Neuropsychopharmacology, 47, 1240–1248. 10.1038/s41386-021-01214-z 34782701 PMC9018815

[brb33545-bib-0024] Joyce, M. K. P. , García‐Cabezas, M. Á. , John, Y. J. , & Barbas, H. (2020). Serial prefrontal pathways are positioned to balance cognition and emotion in primates. The Journal of Neuroscience, 40, 8306–8328. 10.1523/JNEUROSCI.0860-20.2020 32989097 PMC7577604

[brb33545-bib-0025] Korb, A. S. , Hunter, A. M. , Cook, I. A. , & Leuchter, A. F. (2011). Rostral anterior cingulate cortex activity and early symptom improvement during treatment for major depressive disorder. Psychiatry Research, 192, 188–194. 10.1016/j.pscychresns.2010.12.007 21546222 PMC3152489

[brb33545-bib-0026] Mayberg, H. S. , Brannan, S. K. , Mahurin, R. K. , Jerabek, P. A. , Brickman, J. S. , Tekell, J. L. , Silva, J. A. , McGinnis, S. , Glass, T. G. , Martin, C. G. , & Fox, P. T. (1997). Cingulate function in depression: A potential predictor of treatment response. Neuroreport, 8, 1057–1061. 9141092 10.1097/00001756-199703030-00048

[brb33545-bib-0027] Montgomery, S. A. , & Åsberg, M. (1979). A new depression scale designed to be sensitive to change. The British Journal of Psychiatry, 134, 382–389. 10.1192/bjp.134.4.382 444788

[brb33545-bib-0028] Neumann, N. , & Schulte, R. (1989). Montgomery‐Åsberg‐Depressions‐Rating‐Skala zur psychometrischen Beurteilung depressiver Syndrome. Deutsche Fassung. Erlangen: Perimed‐Fachbuch.

[brb33545-bib-0029] Ogasawara, A. , Ohmura, Y. , & Kuniyoshi, Y. (2020). Reward sensitivity differs depending on global self‐esteem in value‐based decision‐making. Scientific Reports, 10, 21525. 10.1038/s41598-020-78635-1 33299062 PMC7725803

[brb33545-bib-0030] O'Neill, C. , Pratt, D. , Kilshaw, M. , Ward, K. , Kelly, J. , & Haddock, G. (2021). The relationship between self‐criticism and suicide probability. Clinical Psychology & Psychotherapy, 28, 1445–1456. 10.1002/cpp.2593 33847028

[brb33545-bib-0031] Orth, U. , & Robins, R. W. (2013). Understanding the link between low self‐esteem and depression. Current Directions in Psychological Science, 22, 455–460. 10.1177/0963721413492763

[brb33545-bib-0032] Pizzagalli, D. , Pascual‐Marqui, R. D. , Nitschke, J. B. , Oakes, T. R. , Larson, C. L. , Abercrombie, H. C. , & Davidson, R. J. (2001). Anterior cingulate activity as a predictor of degree of treatment response in major depression: Evidence from brain electrical tomography analysis. American Journal of Psychiatry, 158, 405–415. 10.1176/appi.ajp.158.3.405 11229981

[brb33545-bib-0033] Pizzagalli, D. A. (2011). Frontocingulate dysfunction in depression: Toward biomarkers of treatment response. Neuropsychopharmacology, 36, 183–206. 20861828 10.1038/npp.2010.166 PMC3036952

[brb33545-bib-0034] Pizzagalli, D. A. , Webb, C. A. , Dillon, D. G. , Tenke, C. E. , Kayser, J. , Goer, F. , & Trivedi, M. H. (2018). Pretreatment rostral anterior cingulate cortex theta activity in relation to symptom improvement in depression: A randomized clinical trial. JAMA Psychiatry, 75, 547–554. 10.1001/jamapsychiatry.2018.0252 29641834 PMC6083825

[brb33545-bib-0035] Qin, P. M. , & Northoff, G. (2011). How is our self related to midline regions and the default‐mode network? Neuroimage, 57, 1221–1233. 10.1016/j.neuroimage.2011.05.028 21609772

[brb33545-bib-0036] Raichle, M. E. , MacLeod, A. M. , Snyder, A. Z. , Powers, W. J. , Gusnard, D. A. , & Shulman, G. L. (2001). A default mode of brain function. Proceedings of the National Academy of Sciences of the United States of America, 98, 676–682. 10.1073/pnas.98.2.676 11209064 PMC14647

[brb33545-bib-0037] Richter, A. , & Gruber, O. (2017). Influence of ventral tegmental area input on cortico‐subcortical networks underlying action control and decision making. Human Brain Mapping, 39, 1004–1014. 10.1002/hbm.23899 29165901 PMC6866482

[brb33545-bib-0038] Richter, A. , Petrovic, A. , Diekhof, E. K. , Trost, S. , Wolter, S. , & Gruber, O. (2015). Hyperresponsivity and impaired prefrontal control of the mesolimbic reward system in schizophrenia. Journal of Psychiatric Research, 71, 8–15. 10.1016/j.jpsychires.2015.09.005 26522867

[brb33545-bib-0039] Rodríguez‐Cano, E. , Alonso‐Lana, S. , Sarró, S. , Fernández‐Corcuera, P. , Goikolea, J. M. , Vieta, E. , Maristany, T. , Salvador, R. , McKenna, P. J. , & Pomarol‐Clotet, E. (2017). Differential failure to deactivate the default mode network in unipolar and bipolar depression. Bipolar Disorders, 19, 386–395. 10.1111/bdi.12517 28714580

[brb33545-bib-0040] Rosenberg, M. (1965). Society and the adolescent self‐image. Princeton University.

[brb33545-bib-0041] Rupprechter, S. , Romaniuk, l. , Series, P. , Hirose, Y. , Hawkins, E. , Sandu, A.‐L. , Waiter, G. D. , McNeil, C. J. , Shen, X. , Harris, M. A. , Campbell, A. , Porteous, D. , Macfarlane, J. A. , Lawrie, S. M. , Murray, A. D. , Delgado, M. R. , McIntosh, A. M. , Whalley, H. C. , & Steele, J. D. (2020). Blunted medial prefrontal cortico‐limbic reward‐related effective connectivity and depression. Brain, 143, 1946–1956. 10.1093/brain/awaa106 32385498 PMC7296844

[brb33545-bib-0042] Rupprechter, S. , Stankevicius, A. , Huys, Q. J. M. , Series, P. , & Steele, J. D. (2021). Abnormal reward valuation and event‐related connectivity in unmedicated major depressive disorder. Psychological Medicine, 51, 795–803. 10.1017/S0033291719003799 31907081

[brb33545-bib-0043] Schmidtke, A. , Fleckenstein, P. , Moises, W. , & Beckmann, H. (1988). Untersuchungen zur Reliabilität und Validität einer deutschen Version der Montgomery‐Åsberg Depression‐Rating Scale (MADRS). *Schweizer Archiv für Neurologie* . Neurochirurgie und Psychiatrie, 139, 51–65. 2455937

[brb33545-bib-0044] Schultz, W. (2006). Behavioral theories and the neurophysiology of reward. Annual Reviews of Psychology, 57, 87–115. 10.1146/annurev.psych.56.091103.070229 16318590

[brb33545-bib-0045] Sheline, Y. I. , Price, J. L. , Yan, Z. , & Mintun, M. A. (2010). Resting‐state functional MRI in depression unmasksincreased connectivity between networks via the dorsal nexus. Proceedings of the National Academy of Sciences of the United States of America, 107, 11020–11025. 10.1073/pnas.1000446107 20534464 PMC2890754

[brb33545-bib-0046] Trost, S. , Diekhof, E. K. , Zvonik, K. , Lewandowski, M. , Usher, J. , Keil, M. , Zilles, D. , Falkai, P. , Dechent, P. , & Gruber, O. (2014). Disturbed anterior prefrontal control of the mesolimbic reward system and increased impulsivity in bipolar disorder. Neuropsychopharmacology, 39, 1914–1923. 10.1038/npp.2014.39 24535101 PMC4059900

[brb33545-bib-0047] Von Collani, G. , & Herzberg, P. Y. (2003). *Revidierte Selbstwertskala nach Rosenberg (RSES)* [Database record]. *APA PsycTests*. 10.1037/t10526-000

[brb33545-bib-0048] Wagner, G. , Koch, K. , Schachtzabel, C. , Peikert, G. , Schultz, C. C. , Reichenbach, J. R. , Sauer, H. , & Schlösser, R. G. (2013). Self‐referential processing influences functional activation during cognitive control: An fMRI study. Social Cognitive and Affective Neuroscience, 8, 828–837. 10.1093/scan/nss074 22798398 PMC3791071

[brb33545-bib-0049] Wagner, G. , Schachtzabel, C. , Peikert, G. , & Bär, K.‐J. (2015). The neural basis of the abnormal self‐referential processing and its impact on cognitive control in depressed patients. Human Brain Mapping, 36, 2781–2794. 10.1002/hbm.22807 25872899 PMC6869596

[brb33545-bib-0050] Wagner, G. , Sinsel, E. , Sobanski, T. , Köhler, S. , & Marinou, V. (2006). Cortical inefficiency in patients with unipolar depression: An event‐related fMRI study with the Stroop task. Biological Psychiatry, 59, 958–965. 10.1016/j.biopsych.2005.10.025 16458263

[brb33545-bib-0051] Walter, M. , Henning, A. , Grimm, S. , Schulte, R. F. , Beck, J. , Dydak, U. , Schnepf, B. , Boeker, H. , Boesiger, P. , & Northoff, G. (2009). The relationship between aberrant neuronal activation in the pregenual anterior cingulate, altered glutamatergic metabolism, and anhedonia in major depression. Archives of General Psychiatry, 66, 478–486. 10.1001/archgenpsychiatry.2009.39 19414707

[brb33545-bib-0052] Weigand, A. , Gärtner, M. , Scheidegger, M. , Wyss, P. O. , Henning, A. , Seifritz, E. , Stippl, A. , Herrera‐Melendez, A. , Bajbouj, M. , Aust, S. , & Grimm, S. (2022). Predicting antidepressant effects of ketamine: The role of the pregenual anterior cingulate cortex as a multimodal neuroimaging biomarker. International Journal of Neuropsychopharmacology, 25, 1003–1013. 10.1093/ijnp/pyac049 35948274 PMC9743970

[brb33545-bib-0053] Whitton, A. E. , Webb, C. A. , Dillon, D. G. , Kayser, J. , Rutherford, A. , Goer, F. , Fava, M. , McGrath, P. , Weissmann, M. , Parsey, R. , Adams, P. , Trombello, J. M. , Cooper, C. , Deldin, P. , Oquendo, M. A. , McInnis, M. G. , Carmody, T. , Bruder, G. , Trivedi, M. H. , … Pizzagalli, D. A. (2019). Pretreatment rostral anterior cingulate cortex connectivity with salience network predicts depression recovery: Findings from the EMBARC randomized clinical trial. Biological Psychiatry, 85, 872–880. 10.1016/j.biopsych.2018.12.007 30718038 PMC6499696

[brb33545-bib-0054] Wood, J. V. , Heimpel, S. A. , Manwell, L. A. , & Whittington, E. J. (2009). This mood is familiar and I don't deserve to feel better anyway: Mechanisms underlying self‐esteem differences in motivation to repair sad moods. Journal of Personality and Social Psychology, 96, 363–380. 10.1037/a0012881 19159137

[brb33545-bib-0055] Yin, X. , Shen, J. , Jiang, N. , Sun, J. , Wang, Y. , & Sun, H. (2022). Relationship of explicit/implicit self‐esteem discrepancies, suicide ideation, and suicide risk in patients with major depressive disorder. PsyCh Journal, 11(6), 936–944. 10.1002/pchj.580 35996046

[brb33545-bib-0056] Zhang, B. , Li, S. , Zhuo, C. , Li, M. , Safron, A. , Genz, A. , Quin, W. , Yu, C. , & Walter, M. (2017). Altered task‐specific deactivation in the default mode network depends on valence in patients with major depressive disorder. Journal of Affective Disorders, 207, 377–383. 10.1016/j.jad.2016.08.042 27750155

