# Supplementary material for: Task‐induced deactivation dysfunction during reward processing is associated with low self‐esteem in a possible subtype of major depression
Source: Brain Behav. 2024 Jun 14;14(6):e3545. doi: 10.1002/brb3.3545 (PMC11177027; doi:10.1002/brb3.3545)

**Supplementary Table 1: Brain activity negatively correlated with self-esteem scores in MDD patients during reward processing, after controlling for the severity of other depressive symptoms.** Shown are the brain regions whose peak-level BOLD responses to presentation of conditioned reward stimuli correlated negatively with RSES scores, when controlled for MADRS scores. To avoid false negative results, we included all brain regions reaching a minimum p < 0.01 with a minimum cluster size of about 10 voxels.

| **Brain region** | **MNI Coordinates and *T* values** | **Cluster size (number of voxels)** |
| --- | --- | --- |
| **L dorsal striatum** | -12 6 15 (2.80) | 47 |
| **L ventral striatum** | -9 12 6 (2.25) | 47 |
| **R dorsal striatum** | 15 6 21 (2.39) | 14 |
| **R ventral striatum** | 6 9 0 (2.34) | 7 |
| **L dorsal pregenual anterior cingulate cortex** | -6 42 21 (2.53) | 27 |
| **R dorsal pregenual anterior cingulate cortex** | 6 36 21 (2.01) | 27 |
| **L subgenual anterior cingulate cortex** | -9 36 -9 (2.33) | 8 |
| **R anteroventral prefrontal cortex** | 18 57 3 (2.30) | 16 |
| **R dorsal frontomedian cortex** | 3 42 45 (2.25) | 6 |
| R middle temporal gyrus, anterior third | 57 -6 -24 (2.25) | 9 |
| **L middle temporal gyrus, middle third** | -48 -36 -6 (2.17) | 8 |

**Supplementary Figure 2:** **Comparison of brain activity positively (A) and negatively (B) correlated with self-esteem scores in MDD patients during reward processing.** (A) Lower self-esteem scores in MDD patients correlated with reduced reward-related activity in predominantly posterior brain regions including occipito-temporal, (intra)parietal and intraoccipital cortices. (B) In contrast to that, increased reward-related activity associated with lower self-esteem scores was found in anterior prefrontal brain regions. Shown are consecutive axial brain slices covering most of the relevant brain regions from z = 24 until z = -18. To increase visibility of the involved brain regions, the statistical threshold of the t-map was lowered to *p* < 0.05, uncorrected.


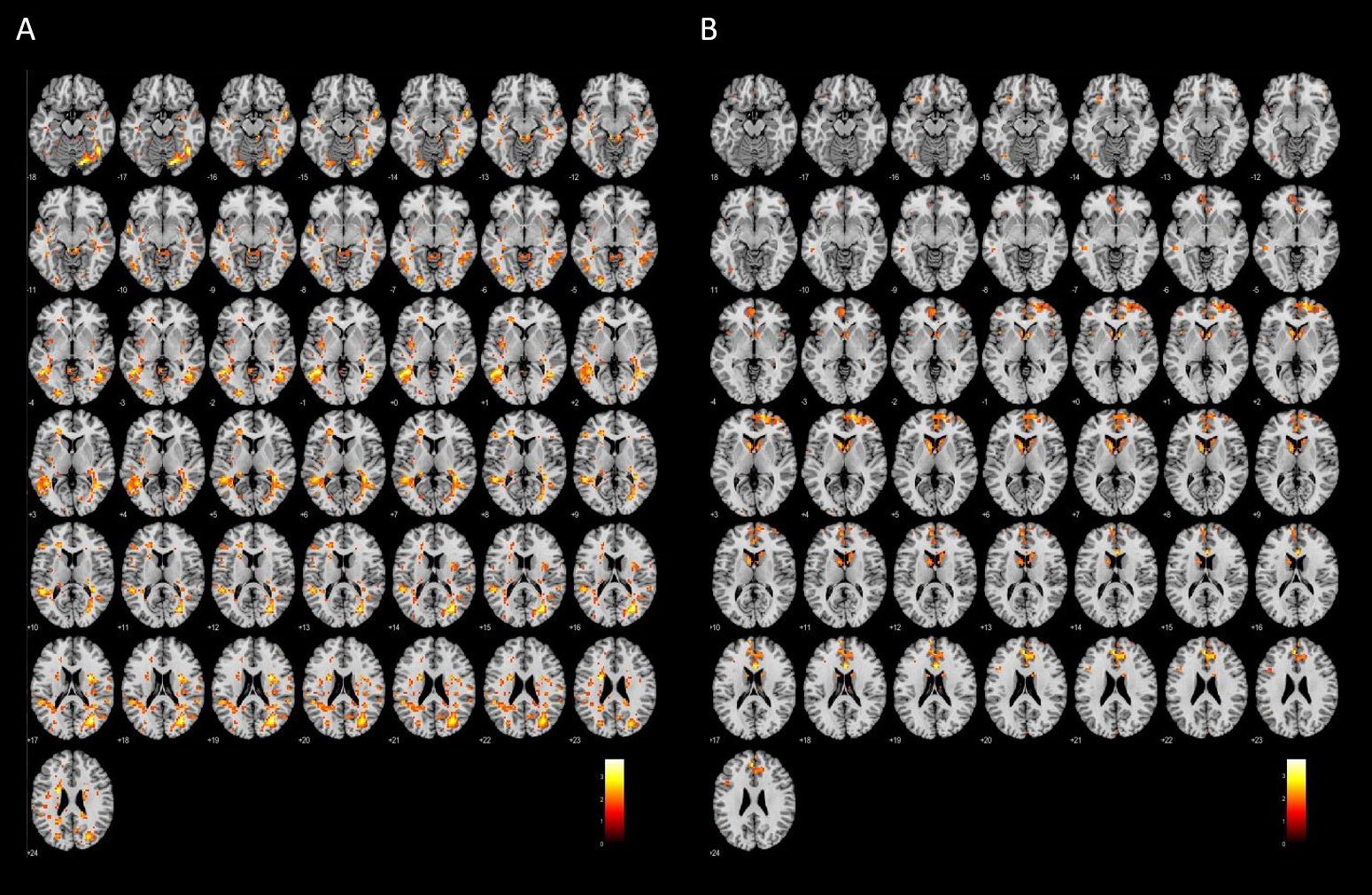

Supplement: Supplementary file 1 — Supporting information [file BRB3-14-e3545-s001.docx]
