# Supplementary figures and images for: Task‐induced deactivation dysfunction during reward processing is associated with low self‐esteem in a possible subtype of major depression
Source: Brain Behav. 2024 Jun 14;14(6):e3545. doi: 10.1002/brb3.3545 (PMC11177027; doi:10.1002/brb3.3545)

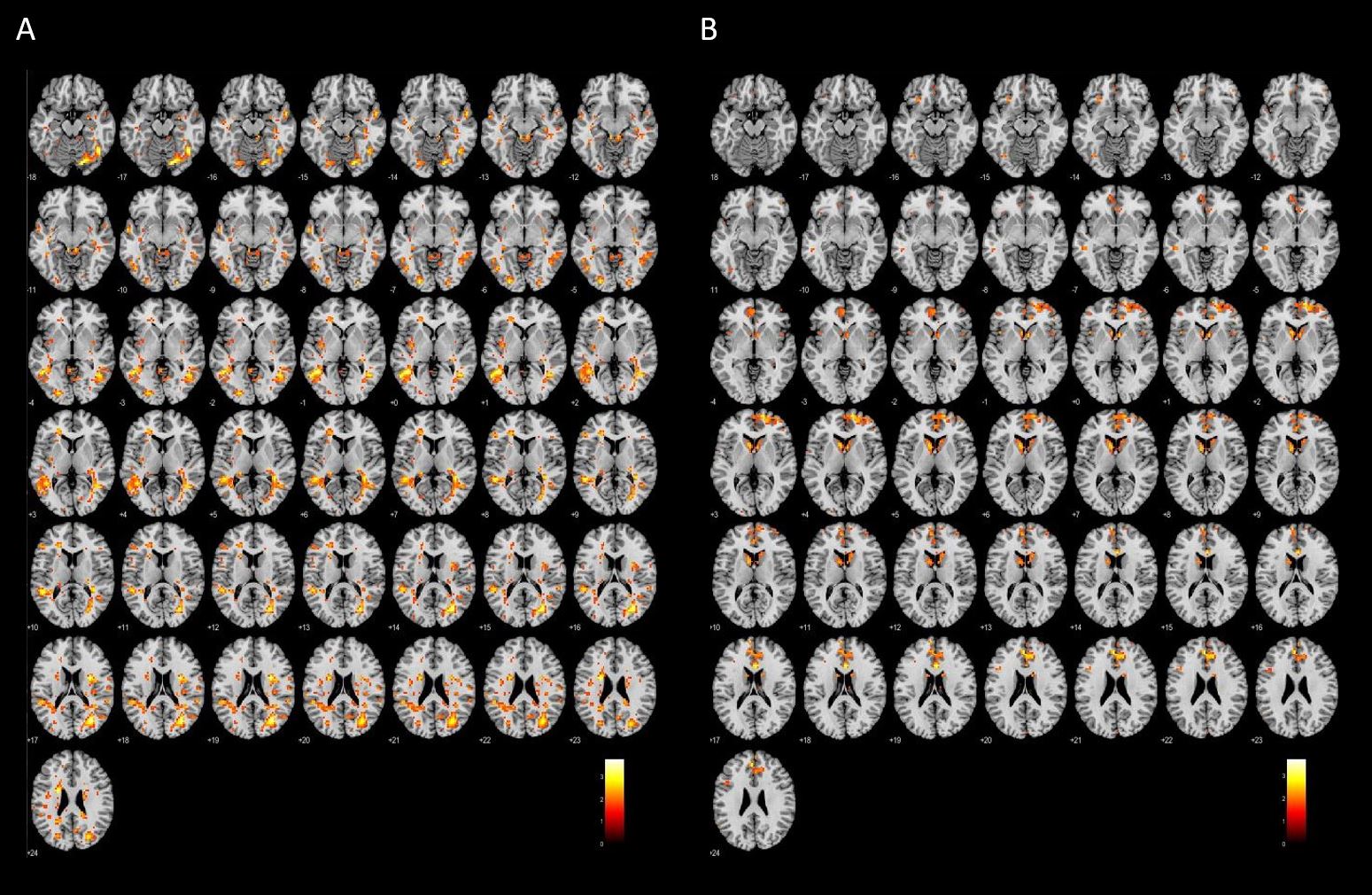

Supplement: Supplementary file 2 — Supporting information [file BRB3-14-e3545-s002.JPG]
